# Long-term outcome of acute type A aortic dissection repair in chronic kidney disease patients

**DOI:** 10.1097/MD.0000000000033762

**Published:** 2023-05-12

**Authors:** An-Hsun Chou, Meng-Ling Hsieh, Yu-Sheng Lin, Dong-Yi Chen, Pao-Hsien Chu, Shao-Wei Chen

**Affiliations:** a Department of Anesthesiology, Chang Gung Memorial Hospital, Linkou Branch, Chang Gung University, Taoyuan City, Taiwan; b Department of Cardiology, Chang Gung Memorial Hospital, Chiayi Branch, Chiayi City, Taiwan; c Department of Cardiology, Chang Gung Memorial Hospital, Linkou Branch, Taoyuan City, Taiwan; d Division of Thoracic and Cardiovascular Surgery, Department of Surgery, Chang Gung Memorial Hospital, Linkou Branch, Chang Gung University, Taoyuan City, Taiwan.

**Keywords:** acute type A aortic dissection, chronic kidney dysfunction, long-term outcome

## Abstract

Preoperative renal dysfunction is associated with mortality in patients with acute type A aortic dissection (ATAAD) repair. However, the long-term outcome of chronic kidney dysfunction (CKD) in ATAAD is unclear. The study aimed to evaluate the long-term outcome of CKD in patients with ATAAD repair. We retrospectively studied patients with ATAAD repair using data from the Taiwan’s National Health Insurance Research Database between July 1, 2004, and December 31, 2013. The outcomes of interest included all-cause mortality, readmission due to any cause, redo aortic surgery, major adverse cardiac and cerebrovascular events, and liver and renal outcomes. There were 3328 patients who received ATAAD repair. These patients were divided into CKD and non-CKD groups. In-hospital mortality in the CKD group was significantly higher than that in the non-CKD group (32.5% vs 18.8%, respectively, odds ratio 2.14, 95% confidence interval [CI] 1.37–3.36). During long-term follow-up, patients with CKD had higher risks of all-cause mortality including in-hospital death (52.6% vs 32.5%; hazard ratio 1.83, 95% CI 1.32–2.55), mortality after discharge (29.7% vs 16.8%; hazard ratio 2.09, 95% CI 1.02–4.29), and readmission rates (67.1% vs 51.6%; subdistribution hazard ratio 2.00, 95% CI 1.43–2.79). However, no significant difference was observed between the dialysis and non-dialysis groups. On the basis of our results, patients with CKD carry a poor long-term outcome after ATAAD repair. Cardiac surgeons should be aware of this condition when dealing with ATAAD repair.

## 1. Introduction

Chronic kidney disease (CKD) is progressively increasing in Taiwan, including end-stage renal disease (ESRD) requiring renal replacement therapy.^[1]^ CKD patients are prone to major cardiovascular comorbidities, including coronary artery disease, valvular disease, and arrhythmia, and may require cardiovascular surgery, which is classified as a high-risk procedure.^[2,3]^ Therefore, the impact of CKD on cardiovascular surgical outcomes is worthy of attention.

Data from United States Renal Data System database revealed poor long-term postoperative outcomes of thoracic aortic interventions for patients with ESRD, regardless of whether an open surgical or endovascular modality is adapted.^[4]^ Additionally, in data from the Japan Adult Cardiovascular Surgery Database, CKD patients were susceptible to high mortality and morbidity after coronary artery bypass grafting, which were dramatically increased in patients on dialysis.^[5]^ In particular, acute type A aortic dissection (ATAAD), a cardiovascular emergency in need of surgical approaches with different complexities, carries critically high mortality and morbidity despite advancing medical management and surgical techniques. Akiyoshi et al^[6]^ conducted a 2-center retrospective cohort of 960 patients undergoing emergent repair of ATAAD and identified increased 30-day mortality and in-hospital death in dialysis patients compared with the non-dialysis group. However, no literature has reported on the long-term outcomes of CKD in the surgical treatment of ATAAD. Therefore, we conducted a nationwide population cohort study to analyze the long-term outcomes of surgical treatment of ATAAD in CKD patients.

## 2. Material and methods

### 2.1. Data source

This national population-based cohort study was conducted using data from the Taiwan National Health Insurance Research Database (NHIRD). The NHIRD is a national population administrative database that contains health insurance claims data from a single-payer National Health Insurance (NHI) program operated by the government, with a coverage rate of > 99%. The Bureau of NHI began issuing all electronic claims data to the public under the NHIRD program in 1999. Disease diagnoses are based on the International Classification of Diseases, Ninth Revision, Clinical Modification (ICD-9-CM). This study was evaluated and approved by the Chang Gung Memorial Hospital Ethics Review Committee (IRB: 104-7990B), and the informed consent was waived because this was a retrospective study.

### 2.2. Identification of patients

**Figure 1.**
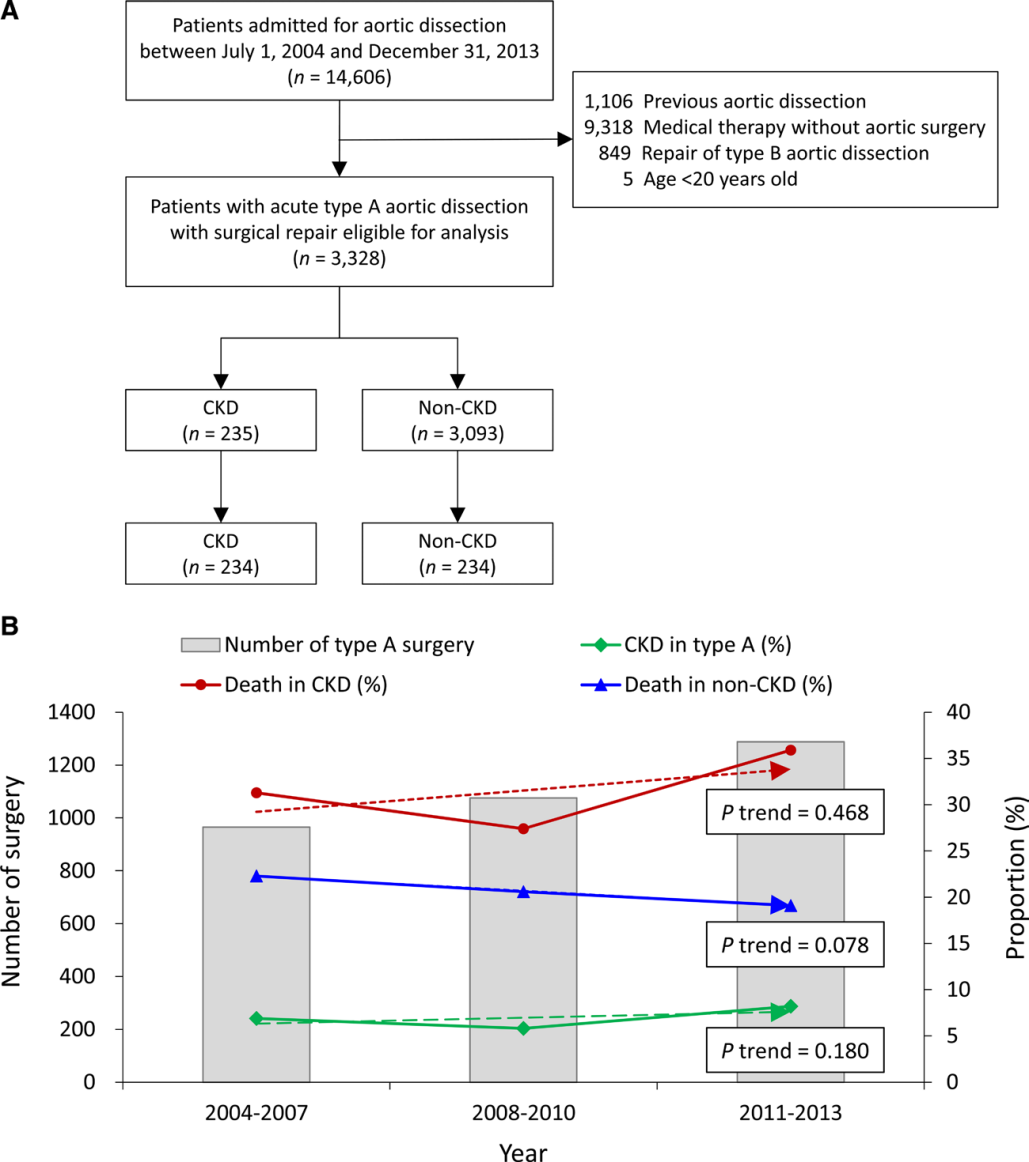
Patient selection (A) and the trend of type A surgery volume, prevalence of CKD, and in-hospital mortality rate of the CKD and non-CKD patients (B). CKD = chronic kidney disease.

From the NHIRD, we obtained the medical claims data sets of inpatients admitted to NHI-contracted hospitals for treatment of ATAAD between July 1, 2004, and December 31, 2013. Patients with a principal diagnosis of aortic dissection (ICD-9-CM: 441.0) were included. Patients who previously had aortic dissection, were treated for type B aortic dissection, received medical therapy instead of surgical intervention and were <20 years old were excluded. In total, 3328 patients who received surgical treatment for ATAAD with ascending aorta replacement, aortic arch replacement, aortic root replacement, or elephant trunk were eligible for analysis. Next, according to the diagnosis of CKD (ICD-9-CM codes: 585.1–585.6), these patients were separated into CKD and non-CKD groups (Fig. 1). To further analyze the severity of CKD on outcomes, we identified ESRD patients by utilizing the Registry for Catastrophic Illness Patient Database.

### 2.3. Covariates and outcomes

Covariates were age, sex, surgical details (extension of aortic surgery and concomitant surgery), comorbidities, hospital level, hospital volume of type A surgery, and surgical year. We utilized the ICD-9-CM diagnostic or procedure codes and the Taiwan NHI reimbursement codes to obtain baseline characteristics and surgical details of the patients based on inpatient claims data. Comorbidities and Charlson Comorbidity Index (CCI) scores were detected using previous inpatient diagnoses, which can be tracked to the year 1997. The disease codes are provided in Table S1, Supplemental Digital Content, http://links.lww.com/MD/I975.

The study outcomes included in-hospital outcomes and late outcomes, which were detected by the ICD-9-CM diagnostic or Taiwan NHI reimbursement codes. Details of the ICD-9-CM diagnostic codes can be found in Table S1, Supplemental Digital Content, http://links.lww.com/MD/I975. Late outcomes were all-cause mortality, readmission due to any cause, redo aortic surgery and major adverse cardiac and cerebrovascular events. Major adverse cardiac and cerebrovascular events were composed of myocardial infarction, heart failure or stroke. Mortality was defined by a withdrawal from the NHI program. Redo aortic surgery was extracted by Taiwan NHI reimbursement codes. The occurrence of acute myocardial infarction, heart failure admission or stroke was defined as the principal diagnosis of admission, where the diagnostic codes have been validated in previous NHIRD studies. Each patient was followed until death or December 31, 2013, whichever came first.

### 2.4. Statistical analysis

A propensity score matching (PSM) method was employed in this study to reduce selection bias between the CKD and non-CKD groups. The propensity score was the predicted probability to be in the CKD group given values of covariates using the logistic regression. The variables selected to calculate propensity score are listed in Table 1, except the follow-up year. Since the enrollment period was long (10 years) in this study, we additionally included the index date as one of the covariates in the calculation of propensity score to consider the possible impact of secular trend. The CCI total score was also not included because CKD is an element of the CCI score. Each patient in the CKD group was matched with a corresponding patient in the non-CKD group. The matching was processed using a greedy nearest neighbor algorithm with a caliper of 0.2 times the standard deviation of the log of the propensity score, with random matching order and without replacement. The quality of matching was checked using the absolute value of standardized difference (STD) between the groups, where a value <0.1 was considered a negligible difference and a value between 0.1 and 0.2 was considered a mild difference.

**Table 1:** Clinical and surgical characteristics of patients with or without CKD before and after propensity score matching.

Variable	Before matching					After matching		
	Total (n = 3328)	CKD (n = 235)	Non-CKD (n = 3093)	STD	CKD (n = 234)		Non-CKD (n = 234)	STD
Demographic								
Age yr	58.9 ± 13.4	61.0 ± 14.8	58.7 ± 13.3	0.16	60.9 ± 14.8		61.2 ± 11.9	−0.02
Age group								
20–60 yr	1772 (53.2)	105 (44.7)	1667 (53.9)	−0.19	105 (44.9)		100 (42.7)	0.04
61–80 yr	1375 (41.3)	115 (48.9)	1260 (40.7)	0.17	114 (48.7)		121 (51.7)	−0.06
>80 yr	181 (5.4)	15 (6.4)	166 (5.4)	0.04	15 (6.4)		13 (5.6)	0.04
Male gender	2275 (68.4)	146 (62.1)	2129 (68.8)	−0.14	146 (62.4)		144 (61.5)	0.02
Comorbidity								
End-stage renal disease (ESRD)	41 (1.2)	41 (17.4)	NA	NA	41 (17.5)		NA	NA
Marfan syndrome	78 (2.3)	1 (0.4)	77 (2.5)	−0.17	1 (0.4)		1 (0.4)	0.00
Hypertension	2287 (68.7)	217 (92.3)	2070 (66.9)	0.67	216 (92.3)		218 (93.2)	−0.03
Diabetes mellitus	346 (10.4)	57 (24.3)	289 (9.3)	0.41	56 (23.9)		54 (23.1)	0.02
Heart failure	149 (4.5)	45 (19.1)	104 (3.4)	0.52	44 (18.8)		43 (18.4)	0.01
Old myocardial infarction	67 (2.0)	12 (5.1)	55 (1.8)	0.18	12 (5.1)		9 (3.8)	0.06
Peripheral arterial disease	115 (3.5)	6 (2.6)	109 (3.5)	−0.06	6 (2.6)		3 (1.3)	0.09
Atrial fibrillation	196 (5.9)	23 (9.8)	173 (5.6)	0.16	22 (9.4)		32 (13.7)	−0.13
Old stroke	258 (7.8)	38 (16.2)	220 (7.1)	0.29	38 (16.2)		31 (13.2)	0.08
Liver cirrhosis	37 (1.1)	3 (1.3)	34 (1.1)	0.02	3 (1.3)		4 (1.7)	−0.04
Coagulopathy	70 (2.1)	7 (3.0)	63 (2.0)	0.06	7 (3.0)		5 (2.1)	0.05
Charlson comorbidity index score (CCI)*	2.1 ± 1.5	4.3 ± 2.0	1.9 ± 1.3	1.45	4.3 ± 2.0		2.4 ± 1.6	1.08
Hospital volume of surgery type A aortic dissection surgery								
1st quartile (1–80)	793 (23.8)	69 (29.4)	724 (23.4)	0.14	69 (29.5)		72 (30.8)	−0.03
2nd quartile (84–144)	836 (25.1)	69 (29.4)	767 (24.8)	0.103	68 (29.1)		58 (24.8)	0.096
3rd quartile (147–206)	920 (27.6)	50 (21.3)	870 (28.1)	−0.16	50 (21.4)		51 (21.8)	−0.01
4th quartile (248–415)	779 (23.4)	47 (20.0)	732 (23.7)	−0.09	47 (20.1)		53 (22.6)	−0.06
Surgery yr								
2004–2007	965 (29.0)	67 (28.5)	898 (29.0)	−0.01	66 (28.2)		69 (29.5)	−0.03
2008–2010	1075 (32.3)	62 (26.4)	1013 (32.8)	−0.14	62 (26.5)		56 (23.9)	0.06
2011–2013	1288 (38.7)	106 (45.1)	1182 (38.2)	0.14	106 (45.3)		109 (46.6)	−0.03
Extension of aortic surgery								
Ascending aorta replacement	2020 (60.7)	133 (56.6)	1887 (61.0)	−0.09	132 (56.4)		140 (59.8)	−0.07
Aortic arch replacement	949 (28.5)	76 (32.3)	873 (28.2)	0.09	76 (32.5)		76 (32.5)	0.00
Aortic root replacement	354 (10.6)	25 (10.6)	329 (10.6)	0.00	25 (10.7)		23 (9.8)	0.03
Elephant trunk	83 (2.5)	9 (3.8)	74 (2.4)	0.08	9 (3.8)		4 (1.7)	0.13
Concomitant surgery								
CABG	347 (10.4)	26 (11.1)	321 (10.4)	0.02	26 (11.1)		16 (6.8)	0.15
Valve replacement	317 (9.5)	19 (8.1)	298 (9.6)	−0.05	19 (8.1)		11 (4.7)	0.14
Propensity score	0.071 ± 0.074	0.145 ± 0.132	0.065 ± 0.064	0.77	0.144 ± 0.130		0.141 ± 0.126	0.02
Follow-up (yr)	2.9 ± 2.8	2.3 ± 2.6	2.9 ± 2.8	−0.24	2.3 ± 2.6		2.9 ± 2.8	−0.22

* Not included in the calculation of propensity score because the CCI score contains CKD.

CKD = chronic kidney disease, CABG = coronary artery bypass graft, NA = not applicable, STD = standardized difference.

In-hospital categorical outcomes between groups were compared by conditional logistic regression analysis, in which the matching pairs were stratified to account for the outcome dependency within the same matching pair. In-hospital continuous outcomes between groups were compared by the linear mixed model, where the 2 patients within the same matching pair were considered to be correlated. Regarding time to event outcomes, the mortality rates between groups were compared by a Cox proportional hazard model. The incidences of nonfatal outcomes between groups were compared by the Fine and Gray subdistribution hazard model, which considered death as a competing risk. The correlation among patients within the same matching pair was accommodated by using the robust standard error (which was known as a marginal model) in the survival analyses. Study group (CKD vs non-CKD) was the only explanatory variable. In a further subgroup analysis of comparing groups of renal function (non-CKD, non-dialysis, and dialysis) on all-cause mortality, we performed a Cox model with multivariable adjustments for age, sex, extension of aortic surgeries and concomitant surgeries. To investigate the risk factors of all-cause mortality, univariable analyses (*t* test or the chi-square test) were performed and further introduced those variables with a *P* value < 0.2 in the univariable analyses into the multivariable Cox analysis with backward selection. A 2-sided *P* value < .05 was considered to be statistically significant, and no adjustment of multiple testing (multiplicity) was made in this study. All statistical analyses were performed using SAS version 9.4 (SAS Institute, Cary, NC), including the procedures of “psmatch” for propensity score matching and “phreg” for survival analysis.

## 3. Results

### 3.1. Epidemiology of CKD in type A surgery

Figure 1B depicts the trend of type A surgery volume, prevalence of CKD, and in-hospital mortality rate of CKD patients. There was an increase in type A surgery across the years. However, the prevalence of CKD and the in-hospital mortality rate in the CKD and non-CKD groups were not changed across the study periods.

### 3.2. Characteristics of patients

Before matching, patients with CKD were older, were less likely to be male, had a lower prevalence of Marfan syndrome, had a higher prevalence of hypertension, diabetes mellitus, heart failure, myocardial infarction, atrial fibrillation, and stroke, had higher Charlson Comorbidity Index scores, were more likely to receive surgery in hospitals with a low volume of type A surgery, were more likely to receive surgery in the recent years and had a short follow-up duration (all the absolute STD values > 0.1). After matching, there were no substantial differences in the baseline characteristics between the groups (all the absolute STD values < 0.1), except for mild differences in atrial fibrillation, elephant trunk and concomitant coronary artery bypass graft (with absolute STD values ranging from 0.1–0.2) (Table 1).

### 3.3. Operation-related outcomes and in-hospital mortality

Operation-related outcomes and in-hospital mortality were calculated from the matched cohort. In-hospital mortality in the CKD group was significantly higher than that in the non-CKD group (32.5% vs 18.8%, respectively, odds ratio [OR] 2.14, 95% confidence interval [CI] 1.37–3.36). Preoperative CKD was also associated with higher risks of respiratory failure and de novo dialysis. Patients with CKD had higher transfusion rates, longer ICU stays, longer hospital stays and greater in-hospital costs (Table 2).

**Table 2:** Operation-related complications and outcomes.

Variable	Total (n = 468)	CKD (n = 234)	Non-CKD (n = 234)	CKD vs Non-CKD	
				OR or B (95% CI)	*P* value
Categorical parameter					
Cardiogenic shock and needed MCS	40 (8.5)	25 (10.7)	15 (6.4)	1.71 (0.89, 3.31)	.109
Respiratory failure	99 (21.2)	65 (27.8)	34 (14.5)	2.55 (1.52, 4.28)	<.001
New onset stroke	53 (11.3)	25 (10.7)	28 (12.0)	0.88 (0.49, 1.57)	.655
New onset ischemic stroke	50 (10.7)	24 (10.3)	26 (11.1)	0.91 (0.50, 1.67)	.758
New onset hemorrhagic stroke	4 (0.9)	2 (0.9)	2 (0.9)	1.00 (0.14, 7.10)	1.000
Reexploration for bleeding	36 (7.7)	23 (9.8)	13 (5.6)	1.91 (0.92, 3.96)	.082
Dialysis (de novo dialysis)	104 (22.2)	77 (32.9)	27 (11.5)	3.78 (2.25, 6.35)	<.001
Sepsis	39 (8.3)	23 (9.8)	16 (6.8)	1.58 (0.77, 3.26)	.213
Deep wound infection	16 (3.4)	7 (3.0)	9 (3.8)	0.78 (0.29, 2.09)	.618
In-hospital mortality	120 (25.6)	76 (32.5)	44 (18.8)	2.14 (1.37, 3.36)	.001
Continuous parameter					
Packed red blood cell	11.9 ± 13.6	14.1 ± 17.4	9.6 ± 7.5	4.53 (2.10, 6.95)	<.001
Fresh frozen plasma	10.7 ± 14.2	12.6 ± 17.9	8.7 ± 8.9	3.89 (1.34, 6.44)	.003
Platelet	22.0 ± 26.0	24.8 ± 28.7	19.3 ± 22.6	5.50 (0.95, 10.05)	.018
ICU duration (days)	12.5 ± 14.8	14.8 ± 17.5	10.3 ± 11.1	4.44 (1.83, 7.06)	<.001
In-hospital stay	28.1 ± 26.3	32.9 ± 31.1	23.3 ± 19.3	9.55 (4.91, 14.19)	<.001
In-hospital cost (NTD × 10^4^)	68.1 ± 45.7	78.4 ± 54.2	57.7 ± 32.1	20.64 (12.81, 28.48)	<.001

CKD = chronic kidney disease, B = regression coefficient, CI = confidence interval, ICU = intensive care unit, MCS = mechanical circulatory support, OR = odds ratio, NTD = New Taiwan dollar.

### 3.4. Late outcomes

**Figure 2.**
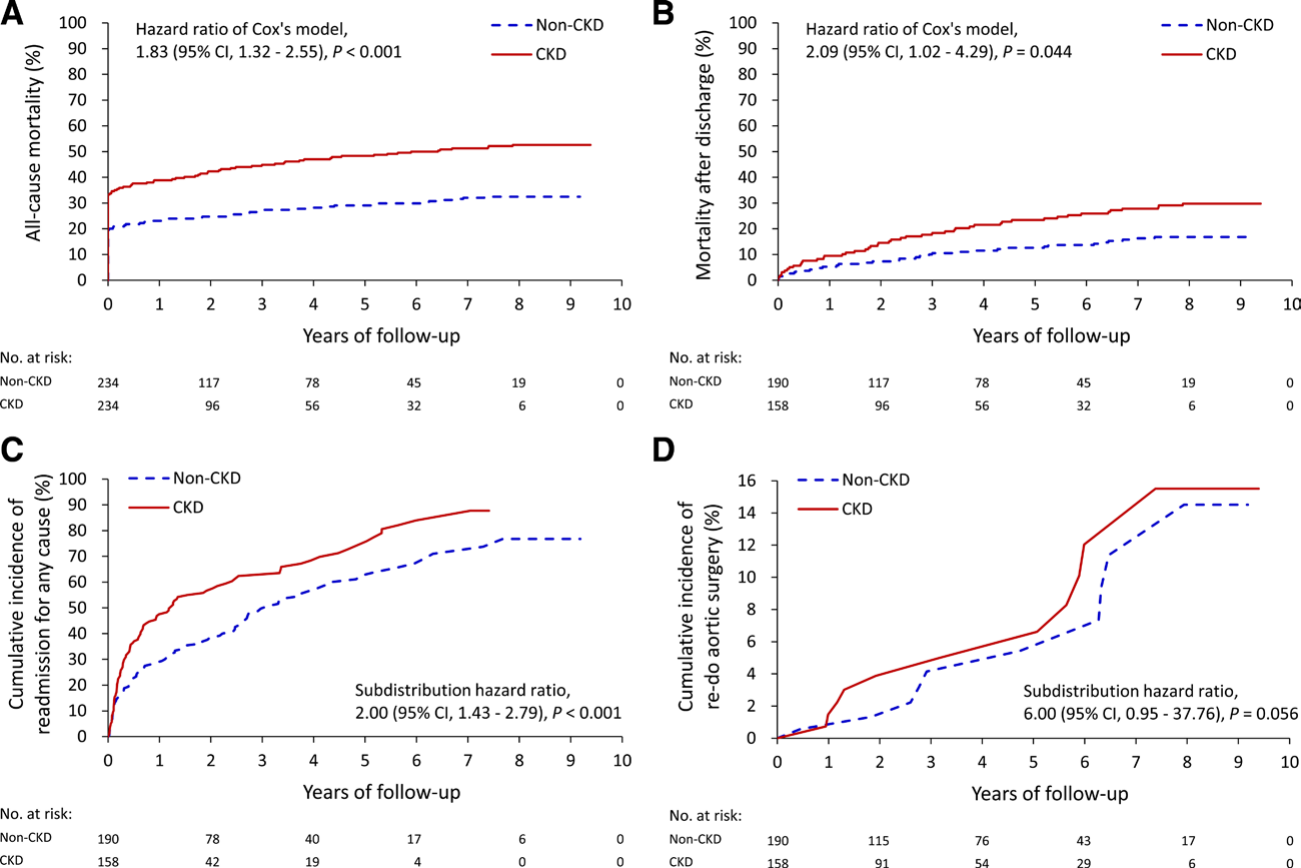
Kaplan–Meier survival curves of all-cause mortality including in-hospital death (A), mortality after discharge, (B) readmission due to any cause, (C) and redo aortic surgery, and (D) of patients with or without CKD in the propensity score matched cohort. CKD = chronic kidney disease.

The results showed that patients with CKD had higher risks of all-cause mortality including in-hospital death (52.6% vs 32.5%; hazard ratio 1.83, 95% CI 1.32–2.55), mortality after discharge (29.7% vs 16.8%; hazard ratio 2.09, 95% CI 1.02–4.29), and readmission rates (67.1% vs 51.6%; subdistribution hazard ratio 2.00, 95% CI 1.43–2.79) (Fig. 2A–C). Preoperative CKD was also associated with borderline higher risks of redo aortic surgery (7.0% vs 5.3%; subdistribution hazard ratio 6.00, 95% CI 0.95–37.76) (Fig. 2D). The results of other secondary late outcomes are provided in Table S2, Supplemental Digital Content, http://links.lww.com/MD/I976.

### 3.5. Subgroup analysis of all-cause mortality

**Figure 3.**
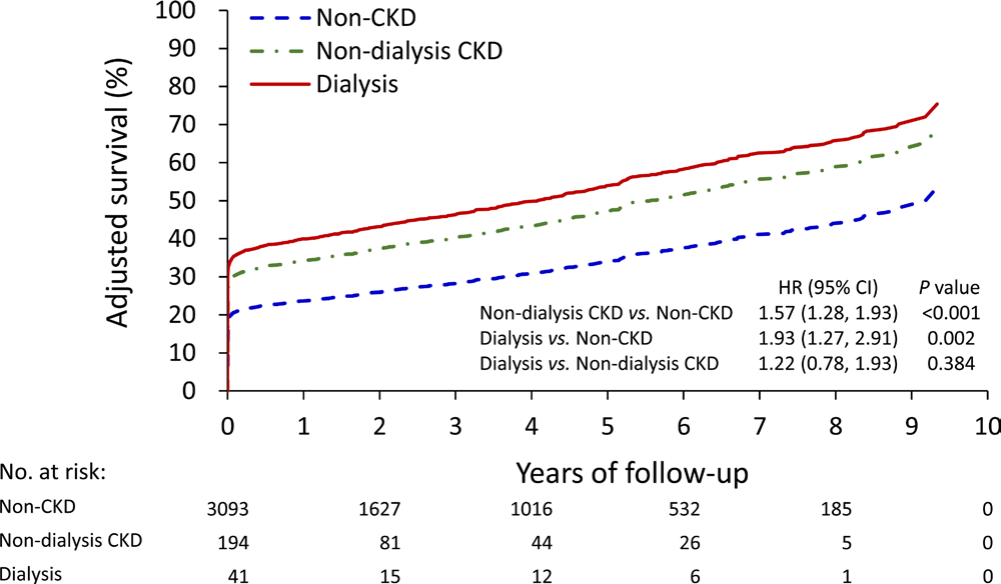
Adjusted all-cause mortality in patients without CKD, those with non-dialysis CKD and those with dialysis in the whole cohort. The data of the y-axis were fitted (adjusted) 1 minus survival curves derived from the multivariable Cox proportional hazard model. CKD = chronic kidney disease.

We conducted a subgroup analysis by comparing all-cause mortality rates in patients without CKD, with non-dialysis CKD and with dialysis using the whole cohort before matching. The analysis was adjusted for age, sex, and surgical details. The results showed that the 2 CKD groups had higher mortality rates than the non-CKD group. However, no significant difference was observed between the dialysis and non-dialysis groups (Fig. 3).

### 3.6. Risk factor analysis for all-cause mortality

Table S3, Supplemental Digital Content, http://links.lww.com/MD/I977 displays the baseline characteristics of patients who died or survived during the entire follow-up in the whole cohort before matching. After introducing those variables with significance < 0.2 into the multivariable Cox model with backward elimination, the result revealed that the following variables were potential risk factors: old age, impaired renal function, stroke, liver cirrhosis, coagulopathy, aortic root replacement, and concomitant coronary artery bypass graft during the index admission. However, the result identified hypertension and higher hospital volume of type A surgery as potential protective factors (Table 3).

**Table 3:** Risk factor analysis of all-cause mortality in the whole cohort.

Variable	Hazard ratio	95% confidence interval	*P* value
Age, per 10 yr	1.27	1.22–1.33	<.001
Renal function			
Non-CKD	1	-	-
CKD without dialysis	1.70	1.38–2.10	<.001
Dialysis	2.07	1.36–3.13	<.001
Hypertension	0.60	0.53–0.68	<.001
Old stroke	1.40	1.15–1.69	<.001
Liver cirrhosis	1.78	1.17–2.72	.007
Coagulopathy	1.78	1.30–2.45	<.001
Hospital volume of type A aortic dissection surgery			
1st quartile	1	-	-
2nd quartile	0.73	0.62–0.85	<.001
3rd quartile	0.77	0.66–0.90	.001
4th quartile	0.68	0.57–0.81	<.001
Aortic root replacement	1.48	1.24–1.77	<.001
Concomitant CABG	1.51	1.28–1.79	<.001

CKD = chronic kidney disease, CABG = coronary artery bypass graft.

## 4. Discussion

In this population-based cohort study, we revealed that CKD patients had a higher incidence of perioperative complications including in-hospital mortality, respiratory failure, de novo dialysis, more blood transfusions, and longer hospital stays than with the non-CKD patients after ATAAD repair. Moreover, in the long-term follow-up period, CKD patients had a higher risk of all-cause mortality and readmission due to any cause, as well as a trend of higher redo aortic surgery.

Despite advances in surgical techniques and postoperative care, operative mortality remains high in both CKD and non-CKD patients who undergo ATAAD repair. Risk adjustment models such as the European System for Cardiac Operative Risk Evaluation (Euro SCORE),^[7]^ Euro SCORE II.^[8]^ and the Society of Thoracic Surgeons database^[9]^ have identified renal dysfunction as a risk factor for poor short-term outcomes in cardiac surgical patients. Okada et al^[10]^ identified severe renal dysfunction, defined as a preoperative eGFR < 30 mL/minutes/1.73 m2, as an independent risk factor for in-hospital death in non-dialysis patients who underwent elective total arch replacement. In a study of patients with CKD who underwent open thoracoabdominal aortic aneurysm repair, considerable risks of operative death and adverse events were observed.^[11]^ Furthermore, the authors found that CKD was an independent predictor of renal failure necessitating dialysis.^[11]^ We agreed that CKD patients had poor short-term outcomes including a higher incidence of in-hospital mortality and perioperative complications during ATAAD surgery. Patients with CKD were older and more likely to have comorbidities than those without CKD, as well as more likely to have worse outcomes. Perioperative management of patients with CKD relies on collaboration between the surgeon, cardiologist, and nephrologist; multidisciplinary intensive care unit rounding is ideal. Additionally, further investigation is needed to improve renal protection during ATAAD repair.

Most of the literature demonstrated that preoperative chronic renal dysfunction reduced long-term survival after cardiac surgery.^[12,13]^ This observation has been confirmed in other study and has established preoperative renal dysfunction as an independent risk factor for late outcome after cardiac surgery.^[14]^ However, the impact of preoperative chronic renal dysfunction on the late outcome of aortic dissection treatment has not been investigated thoroughly. In a study in patients with CKD, it was observed that open thoracoabdominal aortic aneurysm repair carried a worse late outcome than in those without CKD.^[11]^ Our study has added to that work by demonstrating a higher risk of all-cause mortality in CKD patients after ATAAD surgery. In addition, old age, impaired renal function, stroke, liver cirrhosis, coagulopathy, aortic root replacement, and concomitant coronary artery bypass graft were potential risk factors for all-cause mortality after surgical treatment of ATAAD. Multiple focal or diffuse aortic wall calcifications and accelerated atherosclerosis in CKD patients had a chance of aortic dissection and had a trend of higher risk for redo aortic surgery.^[15]^ Moreover, CKD was associated with immune dysregulation and infection, including graft infection.^[16]^ These conditions were associated with higher all-cause mortality and readmission to the hospital after ATAAD repair.

The long-term surgical outcomes of ATAAD in patients with ESRD have not been well investigated. A previous study found that the 6-year survival rate was lower in the dialysis group than in the non-dialysis group in patients who underwent ATAAD repair.^[6]^ In their study, a small number of dialysis patients were compared with non-dialysis patients including those with normal GFR and chronic renal insufficiency without dialysis. In our study, we compared the all-cause mortality in patients without CKD, non-dialysis CKD and dialysis CKD in the follow-up period. The 2 CKD groups had higher mortality rates than the non-CKD group; however, no significant difference was observed between the dialysis and non-dialysis groups. Further large-scale prospective studies are needed to assess the long-term results of ATAAD surgery between dialysis and non-dialysis patients.

## 5. Limitations

This study has several limitations, primarily due to the use of the NHIRD database from Taiwan. First, the NHIRD does not contain disease-specific information including aneurysm-specific data such as size, extent, or morphology. In addition, the diagnosis of CKD was according to the ICD-9 code, instead of measuring the estimated glomerular filtration rate. Different stages of the CKD were unable to classify using ICD-9 code. Because of higher rate of end-stage renal disease in our CKD cohort, and different stages of the CKD were unable to classify using ICD-9 code, we couldn’t explore whether preoperative lower stage CKD will lead to a higher rate of complications and worse long-term outcome after surgery for ATAAD.

Future study was warranted to evaluate the effect CKD stage on outcomes in this population. Second, it is unlikely to assess the reasons for readmission. Nevertheless, because of the link between outpatient clinic and hospital admissions and the NHIRD database, we can overcome the lack of specific accurate data by investigating the exact reasons for readmissions. Third, our study covered a long time period during which surgical techniques and intensive care medicine facilities have improved. And the question of whether high or low volume centers regarding outcomes still remains. To address these questions, we employed a propensity score matching to reduced selection bias between the CKD and non-CKD groups. Furthermore, we could not identify the newly-onset preoperative renal dysfunction complicated by the aortic dissection, or the irreversible deterioration of renal function due to the aortic disease or treatment/surgery, apartment from the CKD which already existed before the onset of aortic dissection.

Finally, although PSM minimized measured confounders in this retrospective study, unmeasured and unknown confounders still exist; therefore, we can only indicate association instead of causation. Despite these limitations, the prevalence and incidence of CKD in Taiwan are relatively high compared with those in other countries,^[17]^ and we believe that the strengths of our study still provide a significant contribution to the analysis of outcomes of ATAAD surgery in CKD patients. However, a prospective randomized trial is still required to assess the long-term results of ATAAD repair in CKD patients.

## 6. Conclusion

In conclusion, patients with CKD carry considerable risks for poor short- and long-term outcomes compared to patients without CKD after ATAAD repair. Patients with CKD face a substantial risk for perioperative complications including in-hospital mortality and adverse events. During long-term follow-up, CKD patients had a higher risk of all-cause mortality and readmission due to any cause, as well as a trend of higher redo aortic surgery. Preoperative counseling, optimizing kidney protection during surgery, and close surveillance of renal function after surgery play an important role in improving the short- and long-term outcomes of ATAAD repair for these patients.

## Acknowledgements

The authors thank Alfred Hsing-Fen Lin and Zoe Ya-Jhu Syu for their assistance in statistical analysis.

## Author contributions

**Conceptualization:** An-Hsun Chou.

**Data curation:** An-Hsun Chou, Meng-Ling Hsieh, Yu-Sheng Lin, Dong-Yi Chen, Pao-Hsien Chu.

**Formal analysis:** An-Hsun Chou, Meng-Ling Hsieh, Yu-Sheng Lin, Dong-Yi Chen, Pao-Hsien Chu.

**Writing – original draft:** An-Hsun Chou.

**Writing – review & editing:** Shao-Wei Chen.

## Supplementary Material






