# Supplementary material for: Long-term outcome of acute type A aortic dissection repair in chronic kidney disease patients
Source: Medicine (Baltimore). 2023 May 12;102(19):e33762. doi: 10.1097/MD.0000000000033762 (PMC10174411; doi:10.1097/MD.0000000000033762)
Supplement: Supplementary file 1 [file medi-102-e33762-s001.pdf]

Supplemental Table 1 ICD-9-CM diagnostic code used in this study

Variable	ICD-9-CM code
Dialysis	585.xx (Catastrophic illness card)
Chronic kidney disease	580.xx–589.xx, 403.xx–404.xx, 016.0x, 095.4x, 236.9x, 250.4x, 274.1x, 442.1x, 447.3x, 440.1x, 572.4x, 642.1x, 646.2x, 753.1x, 283.11, 403.01, 404.02, 446.21
Aortic pathology	
Un-rupture aortic aneurysm	441.xx except 441.0x, 441.1, 441.3, 441.5, 441.6
Rupture aortic aneurysm	441.1, 441.3, 441.5, 441.6
Aortic dissection	441.0x
Traumatic aortic injury	901.0x， 902.0x
Diabetes mellitus	250.xx
Hypertension	401.xx–405.xx
Heart failure	428.xx
Atrial fibrillation	427.31
Coronary artery disease	410.xx–414.xx
Stroke	430.xx–437.xx
Prior myocardial infarction	410.xx, 412.xx
Peripheral arterial disease	440.0x, 440.2x, 440.3x, 440.8x, 440.9x, 443.xx, 444.0x, 444.22, 444.8x, 447.8x, 447.9x
Chronic obstructive pulmonary disease	491.xx, 492.xx, 496.xx
Malignancy	140.xx–208.xx (Catastrophic illness card)
Postoperative infection	038.xx, 9985
Acute myocardial infarction	410.xx

ICD-9-CM=International Classification of Diseases, Ninth Revision, Clinical Modification.
