# Supplementary material for: Long-term outcome of acute type A aortic dissection repair in chronic kidney disease patients
Source: Medicine (Baltimore). 2023 May 12;102(19):e33762. doi: 10.1097/MD.0000000000033762 (PMC10174411; doi:10.1097/MD.0000000000033762)
Supplement: Supplementary file 2 [file medi-102-e33762-s002.pdf]

Supplemental Table 2 Detailed information for late outcomes

Variable	Total (n = 348)	CKD (n = 158)	Non-CKD (n = 190)	CKD vs. Non-CKD HR or SHR (95% CI)	CKD vs. Non-CKD P value
Cox model					
Mortality after discharge	79 (22.7)	47 (29.7)	32 (16.8)	2.09 (1.02, 4.29)	0.044
MACCE*	103 (29.6)	59 (37.3)	44 (23.2)	1.44 (0.79, 2.63)	0.230
Competing risk survival model#					
MACCE$	38 (10.9)	16 (10.1)	22 (11.6)	0.43 (0.21, 0.89)	0.023
Acute myocardial infarction	2 (0.6)	2 (1.3)	0 (0.0)	NA	NA
Heart failure	15 (4.3)	8 (5.1)	7 (3.7)	1.00 (0.32, 3.10)	1.000
Stroke	28 (8.0)	11 (7.0)	17 (8.9)	0.42 (0.19, 0.93)	0.032
Ischemic stroke	24 (6.9)	9 (5.7)	15 (7.9)	0.27 (0.10, 0.77)	0.014
Hemorrhage stroke	4 (1.1)	2 (1.3)	2 (1.1)	2.00 (0.33, 11.97)	0.448
Respiratory failure	49 (14.1)	28 (17.7)	21 (11.1)	1.89 (1.04, 3.43)	0.037
Readmission for any cause	204 (58.6)	106 (67.1)	98 (51.6)	2.00 (1.43, 2.79)	<0.001
Re-do aortic surgery	21 (6.0)	11 (7.0)	10 (5.3)	6.00 (0.95, 37.76)	0.056

CKD= chronic kidney disease, HR= hazard ratio, SHR= subdistribution hazard ratio, CI= confidence interval, MACCE= major adverse cardiac and cerebrovascular event, NA=not applicable.

* Anyone of acute myocardial infarction, heart failure, ischemic stroke and mortality after discharge.

$ Anyone of acute myocardial infarction, heart failure and ischemic stroke.

Estimated using Fine and Gray subdistribution hazard model which considered all-cause death as a competing risk.
