# Supplementary material for: Long-term outcome of acute type A aortic dissection repair in chronic kidney disease patients
Source: Medicine (Baltimore). 2023 May 12;102(19):e33762. doi: 10.1097/MD.0000000000033762 (PMC10174411; doi:10.1097/MD.0000000000033762)
Supplement: Supplementary file 3 [file medi-102-e33762-s003.pdf]

Supplemental Table 3 Patient characteristics stratified by death or survivor during the follow up

Variable	Death (n = 1,164)	Survivor (n = 2,164)	P value
Characteristic			
Age, years	62.2±13.9	57.1±12.8	<0.001
Male gender	786 (67.5)	1,489 (68.8)	0.448
Comorbidity			
CKD group			<0.001
Non-CKD	1,040 (89.3)	2,053 (94.9)	
Non-dialysis CKD	101 (8.7)	93 (4.3)	
Dialysis	23 (2.0)	18 (0.8)	
Marfan syndrome	17 (1.5)	61 (2.8)	0.014
Hypertension	700 (60.1)	1,587 (73.3)	<0.001
Diabetes mellitus	128 (11.0)	218 (10.1)	0.406
Heart failure	77 (6.6)	72 (3.3)	<0.001
Old myocardial infarction	32 (2.7)	35 (1.6)	0.027
Peripheral arterial disease	47 (4.0)	68 (3.1)	0.177
Atrial fibrillation	74 (6.4)	122 (5.6)	0.400
Old stroke	125 (10.7)	133 (6.1)	<0.001
Liver cirrhosis	23 (2.0)	14 (0.6)	<0.001
Coagulopathy	41 (3.5)	29 (1.3)	<0.001
Hospital volume of surgery type A aortic dissection surgery			<0.001
1st quartile (1-80)	329 (28.3)	464 (21.4)	
2nd quartile (84-144)	278 (23.9)	558 (25.8)	
3rd quartile (147-206)	321 (27.6)	599 (27.7)	
4th quartile (248-415)	236 (20.3)	543 (25.1)	
Surgery year			<0.001
2004-2007	438 (37.6)	527 (24.4)	
2008-2010	380 (32.6)	695 (32.1)	
2011-2013	346 (29.7)	942 (43.5)	
Extension of aortic surgery			
Ascending aorta replacement	661 (56.8)	1,359 (62.8)	0.001
Aortic arch replacement	346 (29.7)	603 (27.9)	0.257
Aortic root replacement	170 (14.6)	184 (8.5)	<0.001
Elephant trunk	27 (2.3)	56 (2.6)	0.636
Additional surgery			

Variable	Death (n = 1,164)	Survivor (n = 2,164)	P value
CABG	178 (15.3)	169 (7.8)	<0.001
Valve replacement	141 (12.1)	176 (8.1)	<0.001

CKD= chronic kidney disease, CABG= coronary artery bypass graft.
